# The Role of Epigenetics in Resistance to Cisplatin Chemotherapy in Lung Cancer

**DOI:** 10.3390/cancers3011426

**Published:** 2011-03-17

**Authors:** Kenneth J. O'Byrne, Martin P. Barr, Steven G. Gray

**Affiliations:** Trinity College Dublin, Department of Clinical Medicine, Trinity Centre for Health Sciences, St James Hospital, James Street, Dublin 8, Ireland; E-Mails: kobyrne@stjames.ie (K.J.O.); mbarr@stjames.ie (M.P.B.)

**Keywords:** epigenetics, histone, post-translational modification, DNA methylation, epigenetic modifiers, cisplatin, NSCLC

## Abstract

Non-small cell lung cancer (NSCLC) is the most common cause of cancer related death in the world. Cisplatin and carboplatin are the most commonly used cytotoxic chemotherapeutic agents to treat the disease. These agents, usually combined with drugs such as gemcitabine or pemetrexed, induce objective tumor responses in only 20–30% of patients. Aberrant epigenetic regulation of gene expression is a frequent event in NSCLC. In this article we review the emerging evidence that epigenetics and the cellular machinery involved with this type of regulation may be key elements in the development of cisplatin resistance in NSCLC.

## 1. Introduction

Lung cancer is the cancer with the highest mortality accounting for 28% of all cancer deaths, estimated at 1.3 million deaths worldwide every year [1]. In the USA the incidence and mortality for cancers of the lung and bronchus are expected to be 219,440 and 159,390, respectively, in 2009 [2]. Lung cancer itself is subdivided into two broad categories, non-small-cell lung cancer (NSCLC) and small cell lung cancer (SCLC). NSCLC can then be further divided into three major types, squamous cell carcinoma (SCC), adenocarcinoma and large cell carcinoma. Mortality in lung cancer is high due in part to (a) difficulties in detecting it at an early stage and (b) associated resistance to currently available chemotherapy and radiotherapy regimes [3]. While lung cancer is often considered to be preventable as most cases can be attributed to smoking, approximately 25% of all lung cancers worldwide are not caused by smoking. If considered as a separate entity, lung cancer in never smokers would still rank as the seventh most common cause of cancer death worldwide [3].

**Figure 1.**
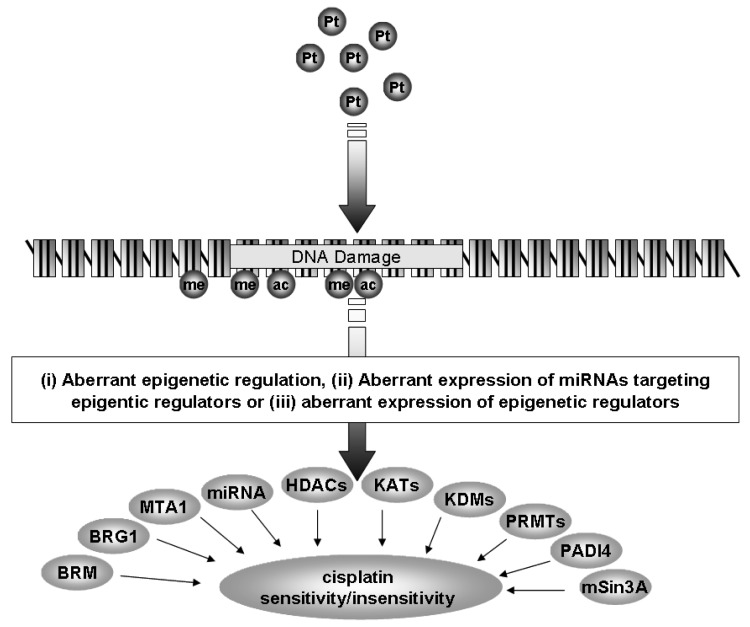
Epigenetics underpinning cisplatin resistance in NSCLC. Diagram summarizing the available evidence linking aberrant epigenetics in the forms of altered gene regulation, or how alterations to the levels of epigenetic modifiers may affect NSCLC sensitivity to cisplatin chemotherapies.

Currently, the standard of care for NSCLC includes treatment with a platinum-based chemotherapy regimen [4]. However, many patients do not benefit from this treatment and tumors often develop resistance to platinum based therapy. In the following review we shall discuss how epigenetics, a specialized form of gene regulation, and the cellular machinery involved with this regulation may be of critical importance in the development of resistance to cisplatin in NSCLC (Figure 1).

## 2. Epigenetics

A modern definition of epigenetics is considered to be stable and heritable changes in gene expression which are not due to changes in the primary DNA sequence. Current known epigenetic mechanisms involve the following: DNA CpG methylation, histone post-translational modifications (PTMs), gene imprinting and non-coding RNA (ncRNA).

### 2.1. miRNAs

miRNAs are specialized forms of ncRNA. They consist of small, approximately 22 nucleotide ncRNAs that regulate gene expression through posttranscriptional silencing of target genes, by binding to complementary sequences on target messenger RNA transcripts (mRNAs), resulting in either mRNA degradation or translational repression and gene silencing. Their primary roles are to regulate the self-renewal, differentiation, and division of cells and their levels are frequently altered in cancer [5]. This differential expression has proven useful to distinguish between small cell lung cancer (SCLC) and non-small cell lung cancer (NSCLC) [6], histological subtypes of NSCLC (squamous *versus* adenocarcinomatous) [7-9], as blood based (plasma or serum) biomarkers for the identification of NSCLC [10,11], prognosis [8,12,13] and for the identification of those miRNAs associated with NSCLC tumorigenesis [14]. It has also been shown that miRNAs can be epigenetically regulated [15,16], and that a specific set of miRNAs can directly regulate the epigenetic machinery (leading to the term epi-miRNAs) [15,16]. In subsequent sections we shall discuss how miRNAs can affect cisplatin resistance in lung cancer either as miRNAs or through epi-miRNA effects (Figure 1).

### 2.2. DNA CpG Methylation and Lung Cancer

DNA can be methylated on cytosine residues. In many cases this methylation takes place on cytosine residues adjacent to guanine residues, also known as CpGs. Methylation of CpG sites within gene promoters can lead to transcriptional repression, a feature found for important genes such as tumor suppressors in a number of human cancers. The importance of DNA methylation in the development of lung cancer was recently demonstrated when it was shown that the transformation efficiency for immortalization of normal bronchial epithelial cells could be enhanced by low dose exposure to carcinogens. The mechanism underpinning this involved hypermethylation of 5–10 genes due to elevated expression of DNA methyltransferase 1 (DNMT1). Ablation of DNMT1 was shown to reverse this process. Moreover, stable “knock-down” of DNMT1 prior to carcinogen exposure was sufficient to prevent cellular transformation [17], and it is well established that aberrant DNA CpG methylation is a well frequent event in lung cancer leading to the inactivation/dysregulation of critical genes [18].

A full discussion of the role of aberrant DNA methylation in cancer is beyond the scope of this review and the reader is directed to the following overviews of this topic [19-21]

### 2.3. miRNAs, DNA Methyltransferases and Lung Cancer

Studies have shown that expression of the enzymes responsible for DNA CpG methylation (DNA methyltransferases or DNMTs), are both upregulated and associated with prognosis in lung cancer [22-24]. One particular miRNA family, the miR-29 family (comprising mIR-29a, -29b, and -29c) has been shown to directly target DNMT3A and -3B and indirectly DNMT1 [25-27], In this regard, the mIR-29 family was found to be downregulated in NSCLC and enforced expression of miR-29s in lung cancer cell lines restored normal patterns of DNA methylation, induced reexpression of methylation-silenced tumor suppressor genes, and inhibited tumorigenicity *in vitro* and *in vivo* [25] (Table 1). Other miRNAs identified in other cancer types also target DNMTs include mIR-148a and mIR-152 (DNMT1) [28], and miR-143 (DNMT3a) [29]. Of these mIR-143 has been shown to be both downregulated in NSCLC and associated with smoking status [30] (Table 1), while in effusions taken from lung cancer patients lower levels of cell-free miR-152 were present in effusions taken from patients who were docetaxol resistant compared to effusions taken from patients who were docetaxol sensitive [31] (Table 1/Figure 1)).

### 2.4. Methylated Genes Associated with Sensitivity to Cisplatin Based Therapy

Significant evidence is emerging linking loss of gene expression in NSCLC by DNA CpG methylation with cisplatin resistance. Indeed, pulsed exposure to cisplatin has been shown to result in drug-induced DNA hypermethylation both *in vitro* and *in vivo* [32,33]. Checkpoint kinase 2 (CHK2) was one of the first examples of one such gene whose downregulation by DNA CpG methylation in NSCLC was associated with resistance to standard chemotherapies including cisplatin [34]. Another gene Empty Spiracles, Drosophila, 2, HOMOLOG OF; (EMX2) has also been shown to be dramatically downregulated in lung cancer tissue samples by methylation of its promoter, and restoration of EMX2 gene expression sensitized lung cancer cells to cisplatin [35]. The serine protease HtrA3 has also been shown to be reduced or completely lost in over 50% of lung cancer cell lines and primary lung tumors from heavy smokers. The loss of HtrA3 expression is due to DNA methylation and results in resistance to both resistance to etoposide and cisplatin [36]. Expression of transglutaminase 2 (TGM2) has been linked to cisplatin resistance in ovarian cancer [37]. Hong and colleagues have now shown that the TGM2 gene is silenced by promoter CpG methylation in approximately one-third of all NSCLC cell lines examined. Cell lines which had loss of TGM2 were more sensitive to cisplatin, and targeting TGM2 using siRNA also resulted in increased sensitivity to this drug [38]. Using microarray technology to compare isogenic parent/resistant cell lines Perona and colleagues identified loss of expression of insulin-like binding protein -3 (IGFBP3) by DNA CpG methylation in cisplatin resistant cells and found a strong correlation between IGFBP3 methylation status and cisplatin response in patients, where methylated promoters were mostly found in patients with cisplatin resistant tumors [39].

The potential for the use of DNA CpG methylation as a biomarker for response to chemotherapy in NSCLC came from a study of circulating serum DNA, where methylation-dependent transcriptional silencing of 14-3-3sigma, a major G2-M checkpoint control gene, was found to be a predictor for longer survival in cisplatin-plus-gemcitabine-treated NSCLC patients [40]. The results discussed above for IGFBP-3, TGM2, CHK2, HtrA3 and EMX2 indicate that a pretreatment analysis of these genes in patients prior to chemotherapy may have translational benefit. More recently Rosell and colleagues have shown that in serum DNA taken from patients enrolled in a multicenter, randomized study of customized cisplatin-based chemotherapy in stage IV NSCLC (clinicaltrials.gov.identifier: NCT00174629) who subsequently underwent second-line chemotherapy or treatment with EGFR tyrosine kinase inhibitors (TKIs) that if the gene for checkpoint with forkhead-associated [41] and ring finger (RF) (CHFR) gene was hypo- or unmethylated in patients receiving second-line EGFR (TKIs), this was associated with longer survival [42].

### 2.5. miRNAs and Sensitivity to Cisplatin Based Therapy

As previously discussed, miRNAs have been shown to have altered expression in lung cancer. But miRNAs themselves have also been linked to cisplatin resistance (Figure 1). In the lung cancer cell line A549 the miRNAs miR-181a, miR-181b and miR-630 have been shown to be involved with cellular responses to cisplatin (Table 1) [43,44]. miR-181a was found to enhance cisplatin triggered cell death by inducing apoptosis through Bax oligomerization, mitochondrial transmembrane potential dissipation, and proteolytic maturation of caspase-9 and caspase-3 [44]. miR-181b was found to be downregulated in an isogenic cisplatin resistant A549 cell line (A549/CDDP), and overexpression of this microRNA decreased levels of BCL2 with resultant enhanced sensitivity to cisplatin induced cell death [43]. It is interesting to note that miR-181a has been found to be both significantly downregulated and associated with poor survival in primary NSCLC tissues [30].

mIR-630 was found to block the early manifestations of the DNA damage response (phosphorylation of ATM, histone H2AX and p53), with concomitant induction of p27(Kip1), reductions in rates of cell proliferation and arrest at the G0-G1 phase of the cell cycle as opposed to the late S-G2-M cell cycle arrest normally mediated by cisplatin [44]

The microRNA-200 family plays important roles in regulating epithelial-to-mesenchymal transition [45]. In NSCLC one of these, miR-200c has been shown to be downregulated in NSCLC as a consequence of DNA CpG methylation (Table 1), and restoration of its expression was shown to restore the sensitivity of a resistant cell line to cisplatin and cetuximab [46]. As such it is becoming very clear that miRNAs may play important roles in tumor cell responses to cisplatin.

### 2.6. Predictive and Prognostic Value of HISTONE Post-Translational Modifications in Cancer

Post-translational modifications of histones or the “histone code” have emerged as a major mechanism by which cells regulate gene expression and cellular function. Aberrant histone post-translational modifications (PTMs) have now been shown to have both predictive and prognostic value in many cancers including adult acute lymphoblastic leukemia (ALL) [47], acute myeloid leukemia AML [48], breast cancer [49,50], colorectal cancer [51], gastric cancer [52], glioma [53], hepatocellular carcinoma [54], lymphoma [51], pancreatic cancer [55], prostate cancer [50,56,57], esophageal cancer [58-60], ovarian cancer [61], and renal cell carcinoma [62-65].

Histone PTMs have also been shown to have both predictive and prognostic value in NSCLC [66,67]. Deregulation of some of the enzymes involved with regulating these modifications in a bronchial epithelial cell transformation model suggest that they play important roles in the transformation process [68]. In addition, strong evidence links aberrant expression of epigenetic regulators, in particular histone deacetylases (HDACs) to chronic obstructive pulmonary disease (COPD), a condition with an increased risk of developing NSCLC [69,70].

### 2.7. Aberrant Levels of Histone Modifying Enzymes in NSCLC

Histone PTMs are carried out by several diverse families of proteins. The best studied of these families are the lysine acetyltransferases (KATs), histone deacetylases (HDACs), K-methyltransferases (KMTs) and K-demethylases (KDMs). The expression of many of these enzymes has now been shown to be altered in NSCLC.

#### 2.7.1. HDACs

The family of HDACs is separated into several classes (Classes I-IV) based on their homology to yeast proteins [71,72]. The Class I members comprise HDACs 1-3 and HDAC8, and in NSCLC, elevated levels of HDAC1 mRNA are found in higher stage (Stage III or IV) cancers [69,70,73], while other members of the class I HDACs have also been observed to have altered expression (Table 2, Figure 1) [69,70,73]. Elevated levels of HDAC3 protein are found in 92% of the SCC subtype (Table 2) [69,70,73], and more recently high expression of HDAC3 has been shown to correlate with poor prognosis in the adenocarcinoma subtype of NSCLC (Figure 1) [74].

The Class II family members comprise HDACs 4, 5, 6, 7, 9 and 10. An analysis of this subclass in NSCLC revealed that reduced mRNA expression for each family member occurred in NSCLC (Figure 1) and was associated with poor prognosis and could act as an independent predictor of poor prognosis with HDAC10 having the strongest predictive capacity (Table 2) [75].

Of the Class III family members Sirtuins (Sirts1-7), Sirt1 has been shown to have altered expression in lung cancer, with 46.4% (45/97) of tumors showing an absence or low expression of SIRT1 protein (Table 2), which was linked to poor prognosis [76].

HDACs form large multi-protein complexes to regulate gene expression [77]. mSin3A, a critical component serving as a scaffold on which the multi-component HDAC co-repressor complex assembles, has also been observed to have decreased expression in NSCLC (Table 2) [78].

ATP-dependent SWI/SNF chromatin remodeling complexes members have also been shown to be altered in the lung. In NSCLC cell lines, the SWI/SNF complex has been found to form a larger complex containing neuron-restrictive silencer factor (NRSF) and its co-repressors, mSin3A and CoREST and it has been suggested that deregulation of NRSF-regulated genes in NSCLC could in fact contribute to enhanced tumorigenicity [69,70,73]. Indeed, expression of the SWI/SNF ATPase subunits, BRG1 and BRM (BRG1/BRM), have been shown to be either mutated or lost in approximately 30% of human non-small lung cancer cell lines (Table 2, Figure 1) [69]. In primary NSCLC tumors, 10% had loss of both BRG1 and BRM, correlating with the poorest prognosis [69]. Using multiple tissue arrays 12 core proteins involved with chromatin remodeling machinery were examined in 300 NSCLC samples (150 adenocarcinomas and 150 squamous cell carcinomas). Two distinct clusters emerged: one containing BRM, Ini-1, retinoblastoma, mSin3A, HDAC1, and HAT1, the other BRG1, BAF155, HDAC2, BAF170, and RbAP48 [69]. Positive nuclear BRM (N-BRM) staining correlated with a favorable prognosis in patients with a five year-survival of 53.5% compared with 32.3% for those patients with tumors that were negative for N-BRM (P = 0.015). Copositivity for both N-BRM and nuclear BRG1 had an increased five year-survival of 72% compared with 33.6% (P = 0.013) in patients whose tumors were positive for either, or negative for both markers. In contrast, membranous BRM (M-BRM) staining correlated with a poorer prognosis in adenocarcinoma patients with a five year-survival of 16.7% compared with those without M-BRM staining (38.1%; P = 0.016) [69].

The expression of Metastasis-associated protein 1 (MTA-1) has been shown to be significantly elevated in NSCLC and was found to be associated with both tumor invasiveness and metastasis (Table 2) [79]. Both MTA-1 and MTA-2 have been shown to functionally associate with histone deacetylases [80], suggesting that the overexpression of MTA's may cause aberrant HDAC activity which may be involved with invasiveness and metastasis of NSCLC.

The E2F transcription factor 1 (E2F1) positively regulates cell cycle progression and also functions as a potent inducer of apoptosis, especially when activated by DNA damage. Studies have now identified miR-449a and mIR449b as microRNAs regulated by this transcription factor [81,82]. mIR-449a has now been shown to target both SIRT1 [82] and HDAC1 [83] (Table 1). Furthermore, levels of miR-449a have been shown to be reduced in lung cancers compared to normal lung tissue (Table 1) [84], and this has functionally been associated with an aberrant epigenetic chromatin configuration through histone H3 Lys27 trimethylation [81]. As a consequence of this, the reduction of miR-449a may in part explain why levels of HDAC1 are frequently overexpressed in lung cancer (Table 1) [69,70,73].

#### 2.7.2. K-Acetyltransferases (KATs; Formerly Acetyltransferases)

K-acetyltransferases (formerly known as either histone acetyltransferases or lysine acetyltransferases [85]) play a multitude of roles in the cell, and we have suggested that these enzymes play important roles in lung cancer [69,70]. For instance, in NSCLC the protein levels of K-acetyltransferase KAT3A (formerly CBP) and E2F-1 were found to be significantly higher in the tumor area than in the corresponding normal epithelium (p < 0.001) (Table 2, Figure 1)) [86]. Mutations within KAT3A have also been described in a small proportion of lung cancer patients [87]. KAT13B (or SRC-3) is also emerging as an important acetyltransferase whose expression is altered or important in cancer. The expression of KAT13B has been shown to be overexpressed in 27% of non-small cell lung cancer (NSCLC) patients correlating with poor disease-free (P = 0.0015) and overall (P = 0.0008) survival (Table 2, Figure 1) [88]. In breast cancer, a recently identified splice variant called SRC-3Delta4 has been found to act as an adaptor protein between EGFR and its downstream signaling molecule FAK to coordinately regulate EGF-induced cell migration, and overexpression of this KAT isoform leads to enhanced breast cancer metastasis to the lung [89]. It will be interesting to see if this splice isoform is overexpressed in NSCLC.

#### 2.7.3. K-Methyltransferases (KMTs; Formerly Lysine Methyltransferases)

K-methyltransferases or KMTs (previously known as lysine methyltransferases [85]) function to add methyl groups to lysine residues as mono-, di- or tri- methylation [90]. Polymorphisms and haplotypes in KMTs have been associated with the risk of developing NSCLC. Polymorphisms and haplotypes associated with a reduced risk of NSCLC have been found in KMT6 (EZH2) and KMT8 (RIZ1) [91,92], while in contrast, polymorphisms in KMT1B (SUV39H2) are associated with an increased lung cancer risk (Table 2) [93].

Altered expression of KMTs has also been shown to be important in NSCLC (Figure 1).

In a lung cancer cell line model where bronchial epithelial (NHBE) cells were immortalized by overexpression of telomerase, SV40 large T antigen, and Ras, it was noted that several KMTs had high expression levels. These were KMT1A (SUV39H1), KMT1C (G9a), KMT1E (SETDB1), KMT4 (DOT1L) and KMT6 (EZH2) (Table 2) [68]. Of these, KMT1C (G9a) when expressed in NSCLC cells causes an aggressive phenotype promoting both invasion and metastasis by silencing expression of the cell adhesion molecule Ep-CAM [94], while overexpression of KMT6 (EZH2) has also been linked to both poor prognosis and cancer aggressiveness in NSCLC [95]. Interestingly, mIR-138 has recently been identified as a miRNA that targets KMT6 (EZH2) [96], and in a study of miRNA profiles for never-smoker lung cancers, this miRNA was a uniquely downregulated miRNA compared to tumors derived from smokers [97].

Menin, the product of the Multiple endocrine neoplasia type 1 (MEN1) gene has been shown to associate with various lysine methyltransferases [98], and mice mutated for Men1 develop NSCLC tumors [99]. In association with KMT6 (EZH2), menin has been shown to suppress lung adenocarcinoma cancer formation by repressing the growth factor pleiotrophin [100], a consequence of which is repression of lung cancer cell migration [101].

#### 2.7.4. Arginine Methyltransferases

Histones can not only be methylated on lysines, they can also be methylated on arginine residues [102]. The enzymes involved are grouped into the protein arginine methyltransferase family (PRMTs) with 11 family members identified to date [103]. Various functions for this family of proteins have emerged including signal transduction, mRNA splicing, transcriptional control, protein translocation, and DNA repair [102]. The expression of various PRMTs has been examined in the mouse lung and for all members (PRMT1-7) examined, strong expression of their respective mRNAs was observed [104]. Immunohistochemical analysis identified strong homogeneous staining of PRMT1 in airway and alveolar type II epithelial cells. In contrast, PRMT2, 3, and 5 exhibited intermittent staining, and were localized in the cytosol of nonciliated airway epithelial cells and alveolar epithelial cells, and were notably absent in vascular smooth muscle and endothelial cells [104]. PRMT4 was present in the apical part of airway epithelial cells and in alveolar epithelial type II cells. Under hypoxia, a significant difference in PRMT2 protein expression was observed, whereas no significant expression differences for all other PRMT isoforms [104]. PRMT4 (also known as CARM1) has however, now been shown to be critical for the control of pulmonary epithelial cell proliferation and differentiation. During embryonic development, loss of CARM1 results in hyperproliferation of pulmonary epithelial cells (particularly alveolar type II cells), and the lungs of newborn mice have a substantially reduced airspace compared with their wild-type littermates. Due to this hyperproliferation lungs from mice lacking CARM1 have immature alveolar type II cells and an absence of alveolar type I cells [105]. Altered expression of this PRMT has been observed in prostate and colon cancer [106-108], but has yet to be fully explored in NSCLC. In this regard significantly upregulated expression of both PRMT1 and PRMT6 mRNA has been observed in NSCLC (Table 2, Figure 1) [109]. Furthermore, knockdown of PRMT1 and PRMT6 in three NSCLC cell lines was associated with a significant suppression of cell growth [109].

#### 2.7.5. K-Demethylases (KDMs; Formerly Lysine Demethylases)

KDMs are a large family of proteins that catalyze the removal of mono-, di-, and tri- methyl marks on lysine residues in both histones and non-histone proteins [110,111]. Using cDNA microarray analysis, Hayami *et al.* identified KDM1A (formerly known as LSD1) as being elevated in bladder, lung and colorectal carcinomas (Table 2, Figure 1), and siRNA knockdown of KDM1 resulted in a decrease of various lung and bladder cancer cell lines, whereas overexpression promoted proliferation [112]. KDM5B (also known as JARID1B/PLU-1) has also been identified as being highly elevated in lung tumor tissues (Table 2) compared with corresponding non-neoplastic tissues and siRNA knockdown of KDM5B significantly suppressed the proliferation of cancer cells and increased the number of cells in sub-G1 phase [113].

Hypoxia has also been shown to play a role in KDM5A (JARID1A) activity in the lung bronchial epithelial cell line Beas-2B and NSCLC adenocarcinoma cell line A549. Under hypoxic conditions, total H3K4 demethylase activity is decreased/inhibited, and knockdown of the major H3K4 demethylase identified in Beas-2B, KDM-5, abrogated this effect [114].

Many K-Demethylases contain a specific JumonjiC (JmjC) domain essential for their demethylase activity [110]. A JmjC containing protein called Mineral Dust-Induced Gene (MDIG)/MYC-Induced Nuclear Antigen (MINA) has been shown to be overexpressed in NSCLC and promotes ribosomal RNA (rRNA) expression through demethylation of tri-methyl lysine 9 on histone H3 at the ribosomal RNA promoter (Figure 1) [115]. To our knowledge, this protein has yet to be assigned within the KDM nomenclature.

#### 2.7.6. Arginine Demethylases

Few arginine demethylases have currently been identified. There is one report on the protein JMJD6 demonstrating that it functions to demethylate histone H3 at arginine 2 (H3R2) and histone H4 at arginine 3 (H4R3) [116]. JMJD6 was originally identified as Phosphatidyl Serine Receptor (PSR) [117], which in bronchial epithelial cells and alveolar cells is associated with the phagocytosis of apoptotic eosinophils [118, 119]. No data has yet emerged on the expression of JMJD6 in NSCLC, although the A549 NSCLC cell line has been shown to express JMJD6 [119].

Another mechanism by which arginine methylation is reversed is through a process known as demethylimination where deimination of the methylated arginine gives rise to citrulline [120]. The protein family responsible, peptidylarginine deiminase (PADI) enzymes currently comprises six members PADI1-6, of which PADI4 is capable of catalyzing the conversion of histone arginine methylation to histone citrullination [120]. Significant overexpression of PADI4 has been observed in NSCLC tumors (Table 2, Figure 1) [121], indicating that aberrant regulation of histone arginine methylation may be important in this disease. Furthermore PADI4 has also been shown to interact with HDAC1 [122], another histone modifying enzyme upregulated in NSCLC. The work by Fuks and colleagues suggest that PADI4 and HDAC1 collaborate to generate a repressive chromatin environment [122], indicating that aberrant repression of critical genes may be an important part of lung cancer tumorigenesis

### 2.8. Specialized Histone PTMs Associated with DNA Double Strand Breaks Caused by Cisplatin

One histone PTM associated with DNA damage repair is gamma histone H2AX (gamma-H2AX). Precancerous lesions of the lung were found to contain signs of a DNA damage response, which included the presence of histone H2AX. This has led to the suggestion by the authors that DNA replication stress is a significant factor in cancer development [123]. The gene Tumor Suppressor Candidate 4; (TUSC4), also known as NPRL2 has now been linked directly to cisplatin sensitivity. In a study of 40 NSCLC cell lines expression of NPRL2 was significantly and reciprocally correlated to cisplatin sensitivity [124], and exogenously expression of NPRL2 resulted in a 2- to 3-fold increase in induction of apoptosis of cells treated with cisplatin [124]. NPRL2 and cisplatin result in the regulation of key components of the DNA-damage checkpoint pathway by promoting (a) downstream gamma-H2AX formation *in vitro* and *in vivo* and (b) higher Chk1 and Chk2 kinase activity resulting in higher levels of G2/M arrest in tumor cells through elevated levels of cell cycle checkpoint [125].

### 2.9. Histone Modifying Enzymes and Cisplatin Resistance

In many solid tumors, various histone modifying enzymes have now been linked to resistance to cisplatin. In lung cancer these include the lysine acetyltransferases KAT13D (Clock) [126], KAT5 (Tip60) [127], KAT2B (PCAF) [128] and KAT13B (SRC-3) [88] (Table 3, Figure 1), while SIRT-1 expression has been linked to cisplatin resistance in epidermoid and hepatoma cells [129]. A recent study has linked both KAT5 (Tip60) and HDAC6 as important regulators of lung cancer cell responses to cisplatin (Table 3). The acetyltransferase Tip60 acetylates an important splicing factor SRSF2 on its lysine 52 residue promoting its proteasomal degradation, while HDAC6 abrogates this. In response to cisplatin an acetylation/phosphorylation signaling network regulates both the accumulation of SRSF2 and splicing of caspase-8 pre-mRNA and determines whether cells undergo apoptosis or G(2)/M cell cycle arrest [130]. Expression of CBP/p300-Interacting Transactivator, with GLU/ASP-Rich C-Terminal Domain, 2; (CITED2 ), has been shown to be involved with cisplatin resistance in cancer cell lines by a process dependent upon p53. Chao and colleagues demonstrated that knockdown of CITED2 sensitized cells in p53 positive cells, whereas H1299 cells which are p53 defective had negligible responses to cisplatin. Knockdown of CITED2 induced KAT3A-mediated p53 acetylation (Lys373) preventing ubiquitination and turnover of p53. This resulted in increased levels of the p53 target Bax, and was further increased following cisplatin treatment [131].

### 2.10. BRCA1 and the DNA Damage Response

The Breast Cancer 1 Gene (BRCA1) has two important functions (i) regulation of gene transcription and (ii) the response to DNA damage (DNA Repair) [132]. Indeed BRCA1 acts mainly as a tumor suppressor through transcriptionally regulating genes involved with DNA repair [133]. Loss of BRCA1 expression is a frequent event in NSCLC [134,135]. Studies have now shown that the loss of BRCA1 and BRCA2 expression can be due to epigenetic inactivation via DNA CpG methylation in 18-30% of tumors [134,136].

BRCA1 forms several complexes in response to DNA damage, and is emerging as a critical regulator of genome integrity through its ability to execute and coordinate various aspects of the DNA damage response [137].

BRCA1 has been shown to form a heterodimer with BARD1 to form an ubiquitin E3 ligase activity [138] that plays an essential role in response to DNA damage. Cisplatin has been shown to directly bind to BRCA1 and its transcriptional transactivation activity is dramatically diminished in the presence of multiple cisplatin-damaged DNA sites [139]. Furthermore, when complexed with BARD1, cisplatin treatment results in a significantly reduced E3 ligase activity [140].

One major multi-protein assembly with which BRCA1 has now been associated with is the Mi-2/nucleosome remodeling and deacetylase NuRD complex (Table 3). In response to DNA double strand breaks (DSBs) induced by ionizing radiation, the catalytic subunit of the NuRD complex CHD4, stimulates the formation of ubiquitin conjugates that facilitate the accrual of RNF168 and BRCA1 proteins to promote DSB repair [141,142].

### 2.11. BRCA1 and Sensitivity to Cisplatin

A clear indication that BRCA1 may be associated with sensitivity to cisplatin came from studies of breast cancer in mice. Cells deficient for BRCA1 were sensitive to cisplatin, while restoration of BRCA1 resulted in increased resistance, and xenografts of cells deficient for BRCA1 were more sensitive to cisplatin than those where BRCA1 had been restored [143-145].

In a study of ovarian cancer, of 115 primary sporadic ovarian carcinomas, 39 (34%) had low BRCA1 protein and 49 (42%) had low BRCA2 expression. Restoration of BRCA1 and BRCA2 mediates resistance to platinum chemotherapy in recurrent BRCA1 and BRCA2 mutated hereditary ovarian carcinomas [146].

In lung cancer the first clinical evidence that BRCA1 levels may predict response to cisplatin came from a study of patients treated with Gemcitabine/Cisplatin in the neoadjuvant setting. In this study patients whose tumors had low levels of BRCA1 mRNA had a better outcome than those whose tumors high levels of BRCA1 mRNA [135,147]. Wang *et al.* confirmed that BRCA1 expression levels in metastatic malignant effusions were negatively correlated with sensitivity to cisplatin (Table 3) [148]. Recently, in a prospective non-randomized phase II clinical trial, Rosell and colleagues tested the possibility that BRCA1 could be used to customize treatment of patients with NSCLC. Patients were segregated and treated based on EGFR mutation status and BRCA1 level. Patients with EGFR mutations received erlotinib, and those without EGFR mutations received chemotherapy with or without cisplatin based on their BRCA1 mRNA levels: low, cisplatin plus gemcitabine; intermediate, cisplatin plus docetaxel; high, docetaxel alone. In addition to BRCA1 the authors also examined its interacting partner proteins (RAP80 and Abraxas) for additional prognostic value. From this analysis it was found that patients with both low BRCA1 and low RAP80, had a median survival exceeding 26 months compared to 11 months for patients with low BRCA1 alone. RAP80 was a significant factor for survival in patients treated according to BRCA1 levels (hazard ratio, 1.3 [95% CI, 1–1.7]; P = 0.05) [149].

For patients with high BRCA1 levels, anti-tubulin-containing regimens have emerged as exciting contenders for therapeutic intervention strategies [133]. Indeed in a recent clinical study, NSCLC patients with high BRCA1 mRNA expression were found to benefit more from this type of treatment (8.7 *vs.* 13.0 months) [150].

### 2.12. BRCA1, K-Methyltransferases and Acquired Cisplatin Resistance

BRCA1-deficient mouse mammary tumor cells are selectively sensitive to an inhibitor of EZH2 [151]. EZH2 (also known as KMT6) is the catalytic subunit of Polycomb repressive complex 2 (PRC2), and is a highly conserved histone methyltransferase that targets lysine-27 of histone H3 [152,153]. A study on the expression of this protein in NSCLC found that patients who had high EZH2 expression in tumor cells had a poorer prognosis than patients who had low EZH2 expression in tumor cells for all pathologic stages of NSCLC (P = 0.001), and that high EZH2 expression was correlated significantly with nonadenocarcinoma histology (P = 0.001) [95] (Table 3). This may be due to the aberrant regulation of mIR-101 which has been shown to regulate expression of EZH2 [154] and this miRNA has been found to be downregulated in NSCLC particularly in the squamous cell subtype [155]. As overexpression of EZH2 has been shown to contribute to the development of acquired cisplatin resistance in ovarian cancer cells *in vitro* and *in vivo* [156], then potentially NSCLC patients with either high BRCA1 or EZH2 levels might potentially benefit from treatments with poly(ADP-ribose) polymerase (PARP) inhibitors such as (DZNep), or could potentially be targeted to induce mIR-101.

### 2.13. BRCA1, the p53/p63/p73 Network, DNA Methylation and Cisplatin Resistance

It is well established that one of the transcription factors which BRCA1 associates with is the Tumor Protein p53 (p53) [157]. This protein plays important roles in regulating the cellular response to DNA damage [158], and levels of p53 has been shown to have prognostic value in NSCLC. In the JBR.10 trial which examined 482 patients with completely resected stage IB and II non-small-cell lung cancer (NSCLC) who received four cycles of adjuvant cisplatin plus vinorelbine or observation alone, patients with p53 protein overexpression had a significantly shortened survival [159]. However, wild-type p53 has also recently been shown to be required for the induction of COX-2 in response to cisplatin treatment in NSCLC cell lines [160]. High expression of this inflammatory enzyme has been shown to inhibit chemotherapy-induced apoptosis. This is in contrast to the findings of Shepherd and colleagues where p53 overexpression was a predictive for significantly greater benefit from adjuvant chemotherapy in completely resected NSCLC patients [159].

Other members of the p53 family also may play a role in cisplatin sensitivity. This family of proteins includes Tumor Protein 63 (p63) and Tumor Protein 73 (p73). An indication that these proteins may also be important in cisplatin sensitivity came from a study of “triple-negative” breast cancer tumors, which found that p63 controlled a pathway for p73-dependent cisplatin sensitivity [161]. A link between DNA CpG methylation and these proteins was recently shown in ovarian carcinoma [162]. BRCA1-deficient cells exhibited hypermethylation within a p73 regulatory region, which included the binding site for the p73 transcriptional repressor ZEB1, leading to the abrogation of ZEB1 binding and increased expression of transactivating p73 isoforms (TAp73). Cisplatin chemotherapy induced TAp73 target genes specifically in BRCA1-deficient cells, and knockdown of TAp73 in these cells caused chemoresistance while having little or no effect on BRCA1-expressing tumor cells. In primary ovarian carcinomas, ZEB1 binding site methylation and TAp73 expression correlated with BRCA1 status and with clinical response [162]. ZEB1 is also a master regulator of the epithelial-mesenchymal transition (EMT) and reports have demonstrated that ZEB1 is important for this process in lung cancer through its regulation of many EMT genes including E-cadherin [163-165], and knockdown of ZEB1 results in the suppression of anchorage-independent cell growth of lung cancer cells [166]. It is interesting to note that mIR-200 has been shown to target ZEB1 (Table 1) [45,167,168], and as levels of mIR-200c are known to be decreased in NSCLC [46], it may be important to determine the BRCA1 status of these tumors.

### 2.14. Epigenetic Targeting Therapies and Reversal of Platinum Based Resistance?

A pleiotropic agent which can act as an HDACi (Phenylbutyrate) has been shown to sensitize head and neck cancers to cisplatin by interfering with the Fanconi anemia and BRCA (FA/BRCA) pathway [169].

ZEB1 regulates E-cadherin expression via recruitment of HDACs and several studies have shown that HDACi can both induce E-cadherin and downregulate ZEB1 indicating a potential mechanism to target ZEB1 mediated effects in NSCLC [163,170,171]

Activation of Transcription Factor 3 (ATF-3) [172], and Activation of Transcription Factor 4 (ATF-4) have both been shown to regulate cisplatin resistance [173]. In this regard ATF-4 has been shown to associate with the lysine acetyltransferase KAT13D to regulate this resistance [126], and downregulation of this acetyltransferase confers sensitivity to cisplatin. However, it has also been shown that the HDACi M344 increased the levels of ATF-3 in A549 cells and enhanced the cytotoxic effects of cisplatin in this cancer cell line [174]

In a recent phase II randomized, double-blinded, placebo-controlled study evaluated the efficacy of vorinostat in combination with carboplatin and paclitaxel in patients with advanced-stage NSCLC. The results indicated a response rate for vorinostat of 34% with vorinostat *versus* placebo 12.5% (P = 0.02). There was also a trend although not significant toward improvement in both median progression-free survival (6.0 months *vs.* 4.1 months; P = 0.48) and overall survival (13.0 months *vs.* 9.7 months; P = 0.17) in the vorinostat arm [175].

Curcumin a lysine acetyltransferase inhibitor has also been shown to promote apoptosis in an NSCLC cell line model of multi-drug resistance through downregulation of mIR-186 [176].

As discussed in previous sections, several genes linked to cisplatin resistance in NSCLC have been shown to be repressed or silenced by DNA CpG methylation and cell treatments with DNMTi have been able to reactivate their expression.

## 3. Conclusions

It is clear from the above sections that epigenetics and the cellular machinery involved in regulating epigenetic regulation of gene expression play important roles in NSCLC tumorigenesis and increasing evidence is demonstrating a clear link between epigenetics and cisplatin resistance in this disease. As we continue to unravel the intricacies of the epigenome, we may be able to more effectively target diseases such as NSCLC by identifying those patients who may be able to benefit from platinum based chemotherapies, and perhaps resensitize patients to chemotherapy using epigenetic targeting. Given the relatively small benefits of platinum based chemotherapy, it is incumbent on medical oncologists and translational scientists to identify those patients most likely to benefit from therapy prior to starting treatment. It is clear from the data presented in this review that there is an immediate potential for testing some of the observed genes/miRNAs within the clinical setting to examine their utility in predicting response to cisplatin based therapy. This may lead to the development of a panel of markers or diagnostic tests that will allow cisplatin to be used only in those patients likely to benefit from therapy without exposing those unlikely to benefit to potential side-effects.

## Figures and Tables

**Table 1.** miRNAs known to target epigenetic machinery and cisplatin resistance in non-small-cell lung cancer (NSCLC).

**miRNA**	**Target**	**Altered in NSCLC**	**Ref.**
miR-29a	DNMT1, -3A, -3B	downregulated	[25]
mIR-29b	DNMT1, -3A, -3B	downregulated	[25]
mIR-29c	DNMT1, -3A, -3B	downregulated	[25]
mIR-101	KMT6	downregulated	[155]
mIR-138	KMT6	Reduced in tumors of never-smokers	[97]
mIR-143	DNMT3A	downregulated	[30]
mIR-152	DNMT1	Reduced in docetaxol resistant patients	[31]
mIR-181a	Bax/Bcl-2	NSCLC cell line model Downregulated in NSCLC	[44] [30]
mIR-181b	Bax/Bcl2	NSCLC cell line model	[43]
miR-200c	ZEB1	Downregulated in NSCLC restoration of expression increases sensitivity to cisplatin	[46]
mIR-449a	SIRT1, HDAC1	downregulated	[84]
mIR-630	Blocks DNA Damage Response	NSCLC cell line model	[44]

**Table 2.** Epigenetic modifiers with altered expression in in non-small-cell lung cancer (NSCLC).

**Gene**	**Comments**	**Reference**
DNMT1	Elevated in NSCLC, prognostic	[22-24]
DNMT3A	Elevated in NSCLC, prognostic	[22-24]
DNMT3B	Elevated in NSCLC, prognostic	[22-24]
HDAC1	Elevated in NSCLC	[69,70,73]
HDAC2	Elevated in NSCLC	[69,70,73]
HDAC3	Elevated in NSCLC, linked to poor prognosis	[69,70,73,74]
HDAC4	Reduced in NSCLC, associated with poor prognosis	[75]
HDAC5	Reduced in NSCLC, associated with poor prognosis	[75]
HDAC6	Reduced in NSCLC, associated with poor prognosis	[75]
HDAC7	Reduced in NSCLC, associated with poor prognosis	[75]
HDAC9	Reduced in NSCLC, associated with poor prognosis	[75]
HDAC10	Reduced in NSCLC, associated with poor prognosis	[75]
SIRT1	Reduced in NSCLC, associated with poor prognosis	[76]
mSin3A	Reduced in NSCLC	[78]
BRG1	lost or mutated in a proportion of NSCLC	[69]
BRM	lost or mutated in a proportion of NSCLC	[69]
MTA-1	Elevated in NSCLC	[79]
KAT3A	Elevated in NSCLC, mutated in a small proportion	[86,87]
KAT13B	Elevated in 27% of NSCLC, prognostic	[88]
KMT1B	Polymorphisms associated with increased risk of NSCLC	[93]
KMT6	Polymorphisms associated with reduced risk of NSCLC Overexpression linked to poor prognosis in NSCLC	[92] [95]
KMT8	Polymorphisms associated with reduced risk of NSCLC	[91]
PRMT1	Upregulated mRNA in NSCLC	[109]
PRMT6	Upregulated mRNA in NSCLC	[109]
KDM1A	Elevated in NSCLC	[112]
KDM5B	Elevated in NSCLC	[113]
MDIG/MINA	Putative KDM, Elevated in NSCLC	[115]
PADI4	Elevated in NSCLC	[121]

**Table 3.** Epigenetic Modifiers associated with cisplatin resistance in NSCLC.

**Gene**	**Reference**
KAT3A	[131]
KAT5	[127,130]
KAT2B	[128]
KAT13B	[88]
KAT13D	[126]
HDAC6	[130]
BRCA1/BRCA2 (NuRD)	[135,147,148,149]
KMT6	[156]
